# Corticomuscular Coupling Analysis in Archery Based on Transfer Entropy

**DOI:** 10.3390/e27101024

**Published:** 2025-09-28

**Authors:** Yunrui Zhang, Yue Leng, Xiaozhi Li, Wenjing Zhang, Hairong Yu

**Affiliations:** 1 School of Biological Science and Medical Engineering, Southeast University, Nanjing 210096, China; 220246875@seu.edu.cn (Y.Z.); lengyue@seu.edu.cn (Y.L.); 2 Department of Physical Education, Southeast University, Nanjing 210096, China; 101006120@seu.edu.cn (X.L.); 101010963@seu.edu.cn (W.Z.)

**Keywords:** corticalmuscular coupling, transfer entropy, archery

## Abstract

Studying the information transfer between the brain and muscles during archery can help us to understand the underlying mechanisms of corticomuscular coupling during motor learning. In this study, we recruited 26 novice archers as participants and calculated the transfer entropy (TE) between their EEG and EMG signals during the archery process. This was performed to assess the characteristics of corticomuscular coupling during archery and the impact of a period of archery training on this coupling. The results indicate that information transfer from EEG to EMG in the α and β frequency bands predominates during archery, which may be related to the roles of α and β frequency bands in inhibitory control and the sustained contraction of muscle stability. Additionally, the optimization of brain resource allocation resulting from a period of archery training is primarily reflected in the prefrontal cortex and motor cortex, where the information transfer from EEG to EMG decreases while activation related to inhibitory control increases. The intensity of corticomuscular coupling weakens with an increase in the number of arrows shot, but archery training reduces the impact of fatigue-induced changes on corticomuscular coupling.

## 1. Introduction

Corticomuscular coupling refers to the functional interaction of cortical and muscle activities. It is widely used as a non-invasive measure to characterize sensorimotor integration, capturing the dynamic relationship through which the brain and muscles exchange afferent and efferent information over time. This coupling reflects both online motor control during movement execution and the selectivity of muscle recruitment. [1,2] Prolonged sports training can alter the corticomuscular coupling method [3], and the corticomuscular coupling system adjusts external disturbances during motor tasks [4]. Corticomuscular coupling is particularly important for fine motor skills like archery. Archery requires intense concentration, sustained muscle tension, and precise cortical control. Not only does it require superb athletic skills, but it also places high demands on the psychological resilience of athletes [5]. Therefore, corticomuscular coupling not only reflects the stability of descending motor commands during archery but also reveals how athletes utilize sensory feedback to adjust posture in real time. Existing studies have shown significant differences in β-band brain-muscle coherence networks between expert and elite archers, suggesting that corticomuscular coupling may serve as an important neurophysiological marker for distinguishing skill levels and training adaptations [6]. Therefore, archery serves as an ideal model to explore the neural and muscular interactions, understanding these processes not only contributes to theoretical research in motor control, but also holds potential applications in optimizing training protocols and rehabilitation strategies.

Electroencephalography (EEG) is a neuroscientific technique that measures cortical electrical activity. EEG is non-invasive, portable, cost-effective, and offers high temporal resolution, making it particularly suitable for studying motor skills compared to other neuroimaging methods [7]. Electromyography (EMG) captures the superimposed electrical activity of superficial muscle fibers and underlying nerve activity recorded from the skin surface. It is also non-invasive, economical, portable, and easy to administer, rendering it highly valuable in sports science and motor behavior research [8]. Analyzing the information transfer between EEG and electromyogram EMG signals during physical activities can aid in understanding the functional coupling between the cortex and muscles and the neural mechanisms controlling muscle activity [9]. Therefore, analyzing the information transfer between EEG and EMG signals during archery can provide valuable insights into the characteristics of corticomuscular coupling during motor learning and help explore the underlying mechanisms. Most researches on CMC are also conducted using EEG and EMG signals, as shown in Table 1.

Many methods have been employed to study corticomuscular coupling during motor execution. Recent methods used to quantify corticomuscular coupling are shown in Table 1. Among these approaches, transfer entropy (TE) has emerged as a particularly powerful tool for characterizing directed and nonlinear dependencies. TE is a measure of the directed transfer of information between two time series, allowing observation of both the strength and the direction of coupling between two systems [19]. As an information-theoretic measure grounded in Shannon entropy principles, TE provides rigorous quantification of nonlinear causal relationships between paired neural time series through conditional probability estimation [20]. Shi et al. used TE between consistent functional frequency bands of EEG and EMG to assess corticomuscular coupling characteristics, and results showed that TE is appropriate for coupling analysis [21]. Arunganesh et al. calculated the symbolic TE between EEG and EMG during standing, level walking, stair descending, and so on, the results suggested that TE is capable of describing the coherence and information exchange between the motor cortex and muscles [22]. Therefore, TE is suitable for the quantitative assessment of corticomuscular coupling states during motor activities. Although advanced variants such as multiscale or wavelet TE have been proposed, we adopted classical TE due to its lower computational complexity and greater robustness under limited sample sizes, making it more suitable for the present study.

This study analyzes the TE between EEG and EMG signals in novice archers before and after a period of archery training to explore the characteristics and changes in corticomuscular coupling during archery. The research aims to uncover the underlying physiological mechanisms, providing valuable insights and guidance for archery participants and contributing theoretical foundations for related applied research.

## 2. Materials and Methods

### 2.1. Participants

A total of 26 participants (7 females, 19 males; age 18.7 ± 0.5 years) were recruited for this study, all freshmen from universities taking an introductory archery course. All participants provided written informed consent and were screened to exclude left-handedness, brain diseases, or brain injuries.

### 2.2. Data Collection and Preprocessing

**Figure 1:**
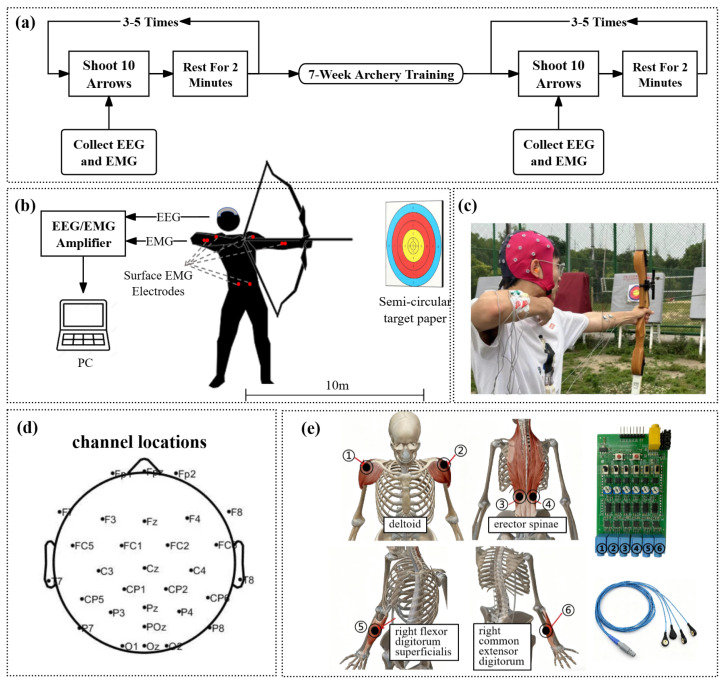
(**a**) Data collection process; (**b**) block diagram of experimental setup; (**c**) experiment scenario; (**d**) locations of the EEG cap channels; (**e**) locations of the specific muscles and EMG equipment.

The experiment was conducted at Southeast University’s outdoor archery range using standardized recurve bows. In accordance with coach recommendations, targets were positioned at the regulation 10-m distance. To observe the effects of a period of archery training on corticomuscular coupling in the participants, EEG and EMG data were collected before (pre-test) and after (post-test) a 7-week archery training program. Training sessions were held once per week, under the guidance of a certified archery coach, each lasting at least 100 minutes. The training environment (including equipment and shooting distance) was kept consistent with the formal testing conditions. Sessions focused on fundamental archery skills such as posture, bow drawing, aiming, and release. While no strict progression schedule was imposed, the coach provided individualized feedback and adjustments based on participants’ performance. During the signal collection process, participants were asked to shoot 10 arrows at a time, with appropriate rest between each set of 10 arrows, performing a total of 30 shooting repetitions in each test. The flow chart is shown in Figure 1a.

The block diagram of the experimental setup and experiment scenario are shown in Figure 1b,c. EEG signals were collected using an ANT Neuro eego amplifier (with dual input for EEG and EMG), a unipolar jackbox, and a 32-channel Waveguard EEG cap according to the standard 10/20 system (${\mathrm{eego}}^{TM}$ mylab 56, ANT Neuro, Germany). The electrode montage included Fp1, Fpz, Fp2, F7, F3, Fz, F4, F8, FC5, FC1, FC2, FC6, T7, M1 (left mastoid), C3, Cz (reference electrode), C4, T8, M2 (right mastoid), CP5, CP1, CP2, CP6, P7, P3, Pz, P4, P8, POz, O1, Oz, and O2, with the ground electrode positioned on the frontal lobe. The channel locations are shown in Figure 1d. The unipolar jackbox was used to collect EMG data from the superficial flexor muscle on the left side, synchronized with the EEG data. EMG signals were collected using six-channel muscle electrical sensors, selecting the left and right deltoid muscles, left and right erector spinae muscles, right flexor digitorum superficialis, and right common extensor digitorum for EMG signal collection [23], The locations of the muscles are shown in Figure 1e. The sampling rates of EEG and EMG are both 1000 Hz.

Both EEG and EMG signals were preprocessed using MATLAB (MathWorks, R2018a, Natick, MA, USA). The preprocessing steps for EEG signals are as follows: (1) A 0.1–45 Hz bandpass filter was initially applied, followed by a 48-52 Hz notch filter to eliminate line noise interference. (2) The average re-reference was applied to transform the data into a reference-free representation. (3) Continuous data were visually inspected to reject artifacts-contaminated segments and defective channels. Channels M1 and M2 (mastoids) are excluded from all the following analyses as they are seriously affected by noise. (4) Independent Component Analysis (ICA) was subsequently conducted to identify and remove components such as ocular and motion artifacts. As for EMG signals, the preprocessing steps are as follows: (1) A Butterworth bandpass filter (40–160 Hz) is applied to remove noise outside the muscle activation range. (2) A 50 Hz notch filter with a 10 Hz bandwidth is used to suppress power line interference. (3) Stationary wavelet transform (db2, level 5) with minimaxi thresholding is applied to reduce in-band noise while preserving signal features.

Based on the participants’ archery scores, the archery performance was divided into three levels: missing the target, low-performance (6–8 rings), and high-performance (9–10 rings). EEG frequency bands were individualized for each participant based on their Individual Alpha Frequency (IAF). For each participant, the IAF was determined by identifying the peak power spectral density within the 8–12 Hz range through spectral analysis of occipital EEG signals (channels O1, O2 and Oz) acquired during resting-state. Occipital electrodes were selected because these sites capture robust posterior alpha peaks that reflect the individual alpha rhythm without being diluted by activity from other brain regions [24]. Subsequent frequency band boundaries were algorithmically defined as follows: a. α band: IAF-2 ∼ IAF + 2 Hz; b. β band: IAF + 3 ∼ 27 Hz; c. γ band: 27 ∼ 45 Hz [25].

### 2.3. TE Between EEG and EMG Signals

**Figure 2:**
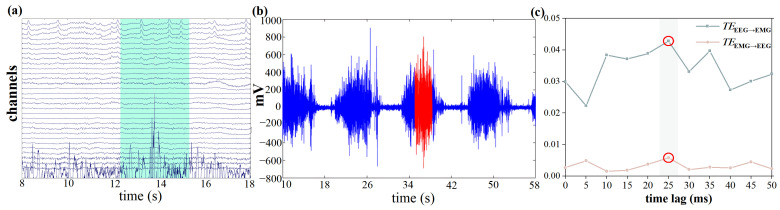
Time window and time lag selection. (**a**) EEG time window selection, where the green-shaded background indicates the 3 s EEG window selected during the shot; (**b**) EMG time window selection, where the red-highlighted segment indicates the 3 s EMG window selected during the shot; (**c**) Time lag selection, where the gray-shaded background and red-circled markers indicate the optimal time delay between EEG and EMG signals.

The EMG signal of the flexor digitorum superficialis reaches its peak at the moment of arrow release; a variety of activations in the brain converge to ultimately determine the release of the arrow. This period contains more activations related to the final decision compared to other time segments [26]. Therefore, a time window of 1.5 s before and after the peak of the left and right flexor digitorum superficialis is selected for calculating the TE between EEG and EMG, as shown in Figure 2a,b. Meanwhile, during the selected time window, participants kept their heads still. This time window was able to minimize the interference caused by motion artifacts during the archery process.

In this study, we calculated the TE from EEG to EMG for time delays ranging from 0 to 50 ms, with an interval of 5 ms, to determine the optimal time lag, the result is shown in Figure 2c. The TE from EEG to EMG reaches its maximum value at the delay of 25 ms, which corresponds to the range of the time lag of information flow from cortex to muscles [27]. Therefore, 25 ms is chosen as the optimal time lag for subsequent analysis.

The calculation method for TE is as follows [28]:

For two processes *X* and *Y*, the information entropy of a random variable *X* is:

(1) $$H\left(X\right)=-\sum_{i=1}^{n}p\left(x_{i}\right)\log p\left(x_{i}\right).$$

The conditional entropy is defined as:

(2) H(Y|X)=H(X,Y)−H(X).

We can interpret H(Y|X) as the uncertainty of *Y* given *X*. Based on this, TE quantifies the reduction in uncertainty of future *Y* when incorporating the past of *X*:

(3) $$TE_{X\to Y|Z}=H\left(Y^{F}|Y^{P},Z^{P}\right)-H\left(Y^{F}|X^{F},X^{P},Y^{P},Z^{P}\right),$$

where $Y^{F}$ represents the past time series relative to $X^{P}$,$Y^{P}$ and $Z^{P}$ is the shifted sequence of *Y* at time delay Δt. Since TE is directional, the net information flow is defined as:

(4) $${\hat{TE}}_{X\to Y}=TE_{X\to Y}-TE_{Y\to X}.$$

A positive ${\hat{TE}}_{X\to Y}$ indicates that *X* is the cause and *Y* the effect.

### 2.4. Statisitical Analysis

All statistical analyses were conducted using IBM SPSS Statistics for Windows, version 27.0 (IBMCorp., Armonk, NY, USA) in this study. Prior to hypothesis testing, data normality was assessed using the Shapiro-Wilk test. As the variables followed a normal distribution (*p* > 0.05 in the Shapiro–Wilk test), different statistical tests were applied based on the study design: Paired-sample *t*-tests were used to compare pre-test and post-test differences within the same participants. Independent-sample t-tests were performed to assess differences between participant groups classified based on archery performance in the same testing session. Spearman correlation coefficients were calculated to examine relationships between measures, as this method captures monotonic associations and is robust to potential deviations from normality. A significance threshold of *p* < 0.05 was applied for both *t*-tests and Spearman correlation coefficients.

Additionally, to ensure that TE values reflected task-related rather than background activities, we compared task-locked epochs (the EMG peak ±1.5 s) with baseline windows of equal length extracted from pre-movement periods with minimal EMG activity. Paired t-tests were conducted for each EEG-EMG pair, and Benjamini–Hochberg false discovery rate (FDR) correction (q < 0.05) was applied across all 180 comparisons (30 EEG channels × 6 muscles). Notably, after FDR correction, 129 pairs (71.7%) remained significant, supporting the task relevance of the observed corticomuscular coupling.

## 3. Results and Discussion

### 3.1. Differences in $TE_{EEG\to \;EMG}$ Across Different Frequency Bands

**Figure 3:**
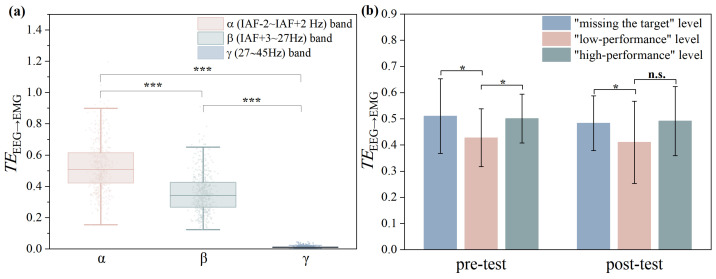
(**a**) Comparison of $TE_{EEG\to EMG}$ across α, β, and γ EEG frequency bands. (**b**) $TE_{EEG\to EMG}$ corresponding to different levels of archery performance. Notes: * indicates significant difference (* means *p* < 0.05, *** means *p* < 0.001). n.s. denotes no statistical significance at the 95 % confidence interval (*p* > 0.05).

Given that neuromuscular adjustments during motor execution are cortically regulated, the study focused more on directed information flow from EEG to EMG. We calculated the differences of mean TE values from EEG to EMG ($TE_{EEG\to EMG}$) of pre- and post-test across the α (IAF-2 ∼ IAF + 2 Hz), β (IAF + 3 ∼ 27 Hz) and γ (27 ∼ 45 Hz) bands, as shown in Figure 3a. However, no significant differences in the distribution of $TE_{EEG\to EMG}$ across the three bands were observed between the pre- and post-test sessions; consequently, we present the mean $TE_{EEG\to EMG}$ values averaged across pre- and post-tests for the subsequent frequency-band analysis. The statistical analysis revealed significant differences in $TE_{EEG\to EMG}$ across α, β and γ frequency bands (*p* < 0.001; |Cohen’s d| = 0.91 for α vs. β, 3.26 for α vs. γ, and 2.74 for β vs. γ). The α and β bands demonstrated a dominant role in corticomuscular information transfer. Notably, the α band exhibited a higher $TE_{EEG\to EMG}$ compared to the β band, suggesting its primary role during the aiming phase in archery.

Existing studies have shown that higher corticomuscular coherence is concentrated in the α and β bands of the EEG [29]. Among these, α band oscillations are the most prominent EEG signals during wakefulness and are highly related to functions such as visual spatial attention and inhibitory control [30,31]. In non-athletes without long-term training, visual input during eye-open conditions induces an increase in the coupling strength of functional cortex-muscle under the α rhythm [32]. β band oscillations are associated with the preparation and execution of voluntary movements [33], as well as the control and maintenance of steady-state muscle contractions [34]. By contrast, γ band oscillations typically appear during rapid movements or brief, high-velocity muscle contractions and reflect high-frequency sensorimotor processing [35]. Archery, however, requires prolonged isometric contractions, which require lower-frequency oscillations for sustaining continuous muscle activation. During archery, the participants are mentally alert, focusing their visual spatial attention on the target, and maintaining steady-state muscle contractions during the aiming phase, thus the information transfer from EEG to EMG in the α and β bands is at a higher level. Meanwhile, the coherence between the low β band (IAF + 3 ∼ 20 Hz) and muscles decreases with movement [36], which further leads to the $TE_{EEG\to EMG}$ in the β band being lower than that in the α band.

### 3.2. The Correlation Between $TE_{EEG\to \;EMG}$ and Archery Performance

Statistical analysis showed that there were significant differences in $TE_{EEG\to EMG}$ corresponding to different archery performance (*p* < 0.05; |Cohen’s d| = 0.56 for “missing the target” vs. “low-performance” in the pre-test, 0.51 for “low-performance” vs. “high-performance” in the pre-test, 0.20 for “missing the target” vs. “low-performance” in the post-test, and 0.47 for “low-performance” vs. “high-performance” in the post-test), as shown in Figure 3b. Spearman’s correlation coefficient was used to evaluate the relationship between $TE_{EEG\to EMG}$ of different muscles and archery performance, and the results are shown in Table 2.

According to Table 1, there was a positive correlation between $TE_{EEG\to EMG}$ and archery performance in the pre-test. While in post-test, the correlation became negative. One possible interpretation is that this change is also related to the optimization of brain resource allocation. Specifically, before training, as novice archers, participants might recruit substantial cortical resources to adjust muscular movements to standardize postures, leading to a positive relationship between $TE_{EEG\to EMG}$ and performance. However, after a period of archery training, the standardization of their postures improved greatly, possibly reducing the need for intensive cortical involvement in motor adjustment and allowing more resources to be devoted to other domains such as visuospatial attention. In this context, excessive reliance on muscle-related control might even interfere with optimal performance, and the positive correlation between $TE_{EEG\to EMG}$ and archery performance no longer exists. However, this explanation should be considered tentative, and further studies are needed to confirm the underlying mechanisms.

During archery, the participation of the motor cortex is fundamental to stable muscle contraction, and a higher archery performance is inseparable from more stable muscle contraction [37]. Therefore, there are significant differences in $TE_{EEG\to EMG}$ corresponding to different levels of archery performance.

### 3.3. Differences in $TE_{EEG\to \;EMG}$ Across Different Brain Regions

**Figure 4:**
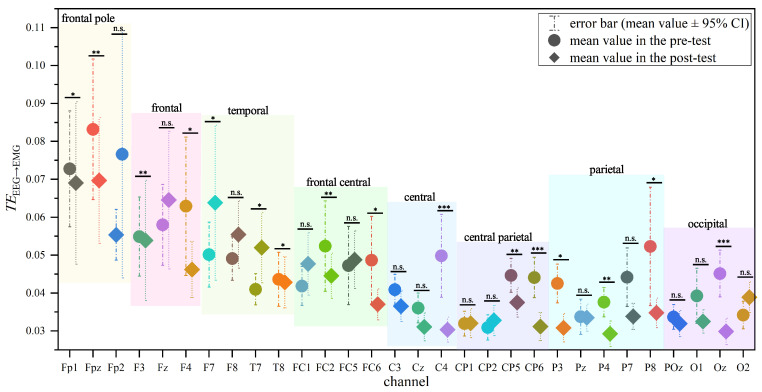
$TE_{EEG\to EMG}$ of different brain regions. Notes: * indicates significant difference (* means *p* < 0.05, ** means *p* < 0.01, *** means *p* < 0.001). n.s. denotes no statistical significance at the 95 % confidence interval (*p* > 0.05).

To observe the differences in information transfer from EEG to EMG across different brain regions, We calculated the mean $TE_{EEG\to EMG}$ from each remaining preprocessed EEG channel to the EMG of six selected muscles. As shown in Figure 4, the $TE_{EEG\to EMG}$ in the frontal lobe region was higher than that in other brain regions, and the difference in $TE_{EEG\to EMG}$ between the frontal region and other brain regions was more pronounced in the pre-test compared to the post-test. The frontal cortex refers to all parts of the frontal lobes outside the primary and secondary motor cortices and is significantly activated during voluntary movement [38]. Additionally, the frontal cortex is extensively involved in important processes, including inhibitory control and attention regulation [39]. The motor cortex, on the other hand, is located in the postcentral gyrus anterior to the central sulcus, where the premotor cortex is responsible for controlling the proximal and trunk muscles of the human body [40]. However, the motor region did not perform a higher $TE_{EEG\to EMG}$ compared to other regions.

Meanwhile, the $TE_{EEG\to EMG}$ in the prefrontal and central regions in pre-test was significantly higher than that in post-test (*p* < 0.05; the specific values of Cohen’s d are provided in Table 3). This reduction may be attributed to a period of archery training leading to increased activation in parts of the prefrontal cortex related to inhibitory control and attention regulation, which in turn results in less information being transmitted to the muscles. Aside from the prefrontal and motor cortices, there were no significant differences in $TE_{EEG\to EMG}$ in other brain regions in post-test compared to pre-test (*p* > 0.05), indicating that the optimization of brain resource allocation brought about by archery training is primarily manifested in the prefrontal and motor cortices.

However, there were no significant differences in TE from EMG to EEG ($TE_{EMG\to EEG}$) across different brain regions, nor were there any significant changes in $TE_{EMG\to EEG}$ after a period of archery training (*p* > 0.05).

### 3.4. The Correlation Between TE and Number of Arrows Shot

**Figure 5:**
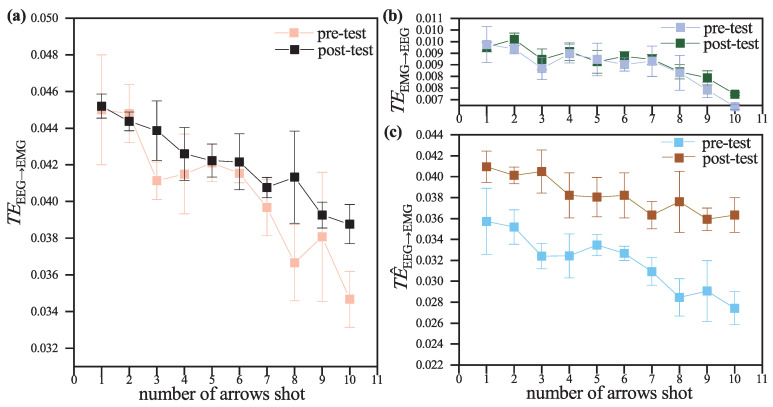
Change in TE with the number of arrows shot. (**a**) $TE_{EEG\to EMG}$; (**b**) $TE_{EMG\to EEG}$; (**c**) ${\hat{TE}}_{EEG\to EMG}$.

To explore the changes in corticomuscular coupling as the archery load increased, we calculated the variations in mean $TE_{EEG\to EMG}$, $TE_{EMG\to EEG}$ and ${\hat{TE}}_{EEG\to EMG}$ (subtracting $TE_{EMG\to EEG}$ from $TE_{EEG\to EMG}$) with the number of arrows shot, the results are shown in Figure 5.

All three indicators exhibited a decreasing trend with the increase in the number of arrows shot, with the post-test decrease being more moderate compared to the pre-test. Within the same test, the decreasing trend of $TE_{EEG\to EMG}$ was more pronounced than that of $TE_{EMG\to EEG}$.

This phenomenon may be due to automatization in the motor learning process. During the acquisition of archery skill, motor control gradually shifts from conscious, goal-directed processes to more automated, procedural patterns of control. This reorganization might be associated with reduced task-related cortical activity and altered corticomuscular coupling [41]. Therefore, the cortical signals could be less predictive of muscle activity, yielding a reduction in $TE_{EEG\to EMG}$. The faster decrease in $TE_{EEG\to EMG}$ compared to $TE_{EMG\to EEG}$ may indicate that the learning of central motor commands occurs before changes in peripheral feedback. This finding supports the view that early learning mechanisms adjust cortical output, while changes in sensory feedback evolve more slowly [42]. Pre-post comparisons of training also show that archery training can help reduce these decreases, which suggests that the efficiency of communication between the brain and muscles is improved after training.

### 3.5. Comparison of TE and Coherence

**Figure 6:**
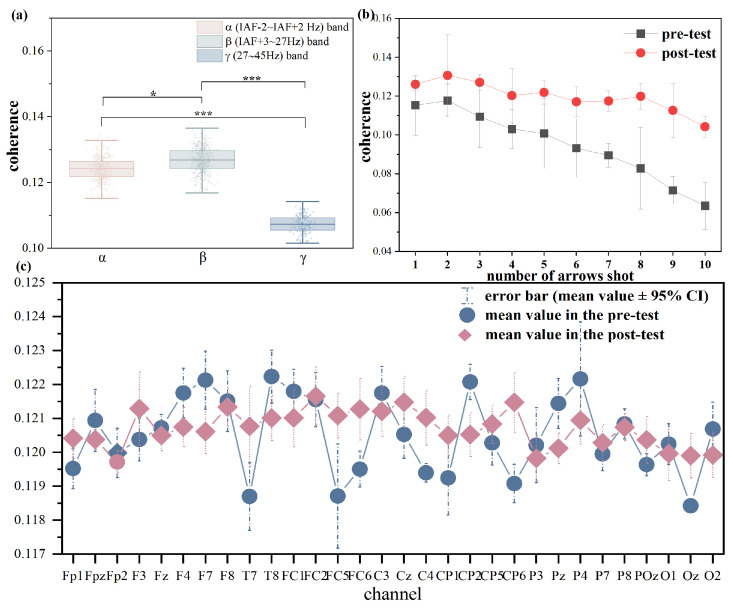
The results of coherence between EEG and EMG. (**a**) Comparison of coherence across α, β, and γ EEG frequency bands; (**b**) Change in coherence with the number of arrows shot; (**c**) Coherence of different brain regions. Notes: * indicates significant difference (* means *p* < 0.05, *** means *p* < 0.001). n.s. denotes no statistical significance at the 95 % confidence interval (*p* > 0.05).

To compare TE with other common methods in corticomuscular coupling, coherence between EEG and EMG (with the same preprocessing steps and time-window selection) is also calculated in this study. The results indicate that both EEG-EMG coherence and TE exhibited comparable frequency-specific modulation across the alpha, beta, and gamma bands and similar task-related changes as the number of shots increased, as shown in Figure 6a,b. However, as shown in Figure 6c, unlike TE, coherence did not reveal regional differences or information-flow direction. One possible reason is that coherence may be blurred by common drive or volume conduction, where widespread EEG rhythms project to multiple electrodes and create spurious similarity across regions, whereas TE can partially remove these influences and reveal true directional flow [43]. In contrast, TE is less sensitive to amplitude variations and can capture non-linear interactions, making it a more robust measure of corticomuscular coupling in this context.

### 3.6. Further Discussion

Despite the constraints imposed by the sample size and the traditional TE method, the findings of this study remain valuable as they highlight consistent trends in corticomuscular coupling changes during archery training. These trends align with established theories of motor learning and cortical control, indicating that the observed phenomena are not random but rather reflect genuine physiological processes. Future studies could further strengthen the reliability of the findings by collecting additional signals and expanding the dataset.

Previous studies on motor skill learning have primarily focused on comparing novices with expert athletes [44]. As an exploratory study focusing on novice archers, these results provide a foundation for future investigations. Future studies involving larger and more diverse participant groups could provide a more comprehensive understanding of corticomuscular coupling across different skill levels.

Furthermore, the above analysis is based on the premise that higher TE represents stronger coupling between signals. Although many articles, including this one, have judged coupling strength through the value of TE, and the results are consistent with other indicators of coupling strength (such as coherence), some articles have explicitly stated that TE cannot provide information on the coupling strength between variables [20]. Therefore, though the analysis results using TE in this article are consistent with the results of other related studies, the relationship between TE and coupling strength is still debatable. Another limitation is the absence of multicenter validation, which may restrict the generalizability of the findings. In addition, only EEG and EMG signals were used, and other complementary modalities like fNIRS or motion capture can be considered to provide a more comprehensive understanding. Future work could also integrate complementary measures such as multiscale entropy, or spectral entropy to provide a more comprehensive characterization, as well as other suggested methods in recent CMC studies, and the reliability of the conclusions can be demonstrated in subsequent studies by introducing other statistical methods into TE.

## 4. Conclusions

In this study, EEG and EMG data were collected from participants during the archery process, the strength and direction of corticomuscular coupling during archery were assessed by calculating the transfer entropy (TE) between the two signals. The findings demonstrate the utility of TE as a quantitative measure for assessing changes in corticomuscular coupling during archery training. By revealing the dominance of α and β frequency bands in corticomuscular information transfer and the effects of training on brain resource allocation, this study offers valuable insights into the neural mechanisms underlying skill acquisition in archery. Importantly, significant training-related changes were observed in $TE_{EEG\to EMG}$ but not in $TE_{EMG\to EEG}$. Such directional asymmetry suggests that archery training primarily strengthens top-down cortical modulation rather than bottom-up muscular feedback. Although limited by sample size and the exclusive focus on novice participants, these results provide a foundation for future research aiming to optimize training protocols and explore applications in sports performance and rehabilitation.

## Figures and Tables

**Table 1:** Recent Studies for corticomuscular coupling (2020–2025).

Year	Author(s)	Modality	Method	Main Findings/Significance
2020 [10]	Liang et al.	EEG + EMG during ankle dorsiflexion	TFMIC (time–frequency maximal information coefficient)	TFMIC demonstrates accurate detection of corticomuscular coupling across diverse functional relationships under low noise; The strength of coupling is notably higher in the α and β bands compared to the γ band.
2021 [11]	Liu et al.	EEG + EMG during force-tracking task	MSTSE (Multiscale Transfer Spectral Entropy)	Corticomuscular coupling strength in the β band is significantly stronger in the cortical-to-muscle direction than in the muscle-to-cortical direction. MSTSE can effectively construct multiscale corticomuscular coupling information.
2021 [12]	Liang et al.	EEG + EMG during steady grip	TDMIC (time-delayed maximal information coefficient)	Muscle fatigue leads to a significant increase in beta-band information flow within corticomuscular coupling and alters the magnitude of information transfer in both directions (cortex to muscle and muscle to cortex).
2021 [13]	Guo et al.	EEG + EMG during a motor control task	MWTE (Multiscale Wavelet Transfer Entropy)	The application of MWTE for detecting information transfer between EEG and EMG can simultaneously capture nonlinear cross-frequency and cross-scale interactions.
2023 [14]	Guerrero-Mendez et al.	EEG + EMG during manipulation tasks involving varying contact surfaces	PBC (Power-Based Connectivity) and MI (Mutual Information)	Compared to the resting state, the anterior deltoid demonstrates the strongest activation and the highest corticomuscular coupling during active object manipulation.
2024 [15]	Roeder et al.	EEG + EMG during overground walking	Multivariate coherence decomposition	During the gait cycle, the trajectories of corticomuscular coupling networks were contracted in older adults, but exhibited specific enhancement in individuals with impaired foot tactile sensation.
2024 [16]	Costa et al.	EEG + EMG during different overground walking balance tasks	coherence spectral analysis	The strength of corticomuscular coupling in proximal muscles increases with both age and the degree of balance impairment; cortical involvement in the neural control of walking balance becomes more pronounced with advancing age.
2025 [17]	Zhao et al.	EEG + EMG during sitting, standing, and free walking	PAC (Phase–amplitude coupling)	Both cortical and corticomuscular β–γ phase–amplitude coupling are modulated by levodopa administration and motor activity. Specifically, low-frequency β–γ corticomuscular phase–amplitude coupling is closely associated with gait dysfunction.
2025 [18]	Hakkak Moghadam Torbati et al.	Magnetoencephalography (MEG) + EMG during isometric pinch task	Pearson correlation, Lyapunov exponent, fractal dimension, and correlation dimension	Nonlinear features capture the intrinsic and stable dynamics of cortical and muscular beta activity, but do not reflect cross-modal similarity.

**Table 2:** Correlation coefficients between $TE_{EEG\to EMG}$ and archery performance in pre- and post-test.

Group	Pre-Test		Post-Test	
	Spearman Correlation Coefficient	* p * Value	Spearman Correlation Coefficient	* p * Value
Left deltoid	0.1711	0.0986	−0.2917	0.0384 *
Right deltoid	0.2294	0.0456 *	−0.0960	0.0998
Left erector spinae	0.0929	0.0310 *	−0.3649	0.0428 *
Right erector spinae	0.1605	0.1601	−0.1395	0.0555
Right flexor digitorum superficialis	0.2729	0.0908	−0.1507	0.0235 *
Right common extensor digitorum	0.1945	0.0280*	−0.2523	0.0438 *

Notes: * indicates significance value (* means *p* < 0.05).

**Table 3:** Effect sizes (Cohen’s d) of the differences in $TE_{EEG\to EMG}$ between pre-test and post-test across different brain regions.

Brain Regions	Frontal		Temporal		Frontal-Central		Central-Parietal		Parietal		Occipital	
	**Channels**	**Cohen’s d**	**Channels**	**Cohen’s d**	**Channels**	**Cohen’s d**	**Channels**	**Cohen’s d**	**Channels**	**Cohen’s d**	**Channels**	**Cohen’s d**
	Fp1	0.39	F7	0.65	FC1	0.35	Cz	0.35	P3	0.68	POz	0.39
	Fpz	0.62	F8	0.39	FC2	0.48	CP1	0.38	Pz	0.42	O1	0.37
	Fp2	−0.08	T7	0.47	FC5	−0.19	CP2	0.24	P4	0.74	Oz	0.62
	F3	0.17	T8	0.18	FC6	0.59	CP5	0.63	P7	0.70	O2	0.44
	Fz	−0.23			C3	0.25	CP6	0.74	P8	0.51		
	F4	0.59			C4	0.67						

## Data Availability

The datasets presented in this article are not readily available because the data are part of an ongoing study. Requests to access the datasets should be directed to the corresponding authors.

## Abbreviations

The following abbreviations are used in this manuscript:

EEG	Electroencephalogram
EMG	Electromyogram
TE	Transfer entropy
$TE_{EEG\to EMG}$	Transfer entropy from EEG to EMG
$TE_{EMG\to EEG}$	Transfer entropy from EMG to EEG
